# Sexual Dysfunction and Its Relationship With Hypogonadism and Myelopathy in Male Patients With X‐Linked Adrenoleukodystrophy

**DOI:** 10.1002/jimd.70121

**Published:** 2025-12-02

**Authors:** Stephanie I. W. van de Stadt, Aimy M. A. Wessel, Mirjam Langeveld, Marc Engelen, Barbara Sjouke

**Affiliations:** ^1^ Department of Pediatric Neurology Emma Children's Hospital, Amsterdam University Medical Centers Amsterdam the Netherlands; ^2^ Department of Endocrinology and Metabolism, Amsterdam UMC Research Institute Gastroenterology, Endocrinology and Metabolism (AGEM), University of Amsterdam Amsterdam the Netherlands; ^3^ Department of Internal Medicine Radboud University Medical Centre Nijmegen the Netherlands

**Keywords:** hypogonadism, myelopathy, sexual dysfunction, testosterone deficiency, X‐linked adrenoleukodystrophy

## Abstract

X‐linked adrenoleukodystrophy (ALD) is a genetic disorder with neurological and endocrine manifestations, including myelopathy and hypergonadotropic hypogonadism. Many patients experience sexual dysfunction but the underlying cause has not been clarified. The aim of this study was to assess the prevalence of sexual dysfunction and its relationship with biochemical hypergonadotropic hypogonadism (i.e., low testosterone and high luteinizing hormone [LH] levels) and/or myelopathy. Male ALD patients from “the Dutch ALD cohort” at the Amsterdam University Medical Centers were questioned regarding sexual functioning, and underwent neurological examination to assess myelopathy. Testosterone, LH, and follicle‐stimulating hormone (FSH) concentrations were analyzed from fasting blood samples. The prevalence of biochemical hypogonadism in the cohort was 4/36 patients (11%), of whom 3 were diagnosed prior to the initiation of this study. Eighteen out of 33 patients (55%), of whom clinical data were collected, experienced sexual dysfunction. Of these patients, 10 (55%) were biochemically eugonadal, 7 (39%) had biochemical subclinical hypogonadism, and 1 had biochemical hypogonadism. Neurological function differed significantly between patients with and without sexual dysfunction, with more severe myelopathy in patients with sexual dysfunction (Expanded Disability Status Scale: 4.8 [IQR 3–6] vs. 0.0 [IQR 0–2]; *p* < 0.001). In conclusion, we showed that half of all adult male ALD patients experienced sexual dysfunction and the majority of these patients had normal testosterone levels. Neurological impairment appears to play a more important role in these complaints than previously appreciated. We recommend discussing sexual dysfunction regularly, in order to start early and adequate treatment.

## 1 Introduction

X‐linked adrenoleukodystrophy (ALD, OMIM #300100) is a genetic metabolic disorder with a broad spectrum of neurological and endocrine manifestations. ALD is caused by pathogenic variants in the *ABCD1* gene, located on the X‐chromosome [1]. Impaired functioning of the peroxisomal membrane protein ALDP results in impaired β‐oxidation of very long chain fatty acids (VLCFA; ≥ C22) and accumulation of VLCFA in plasma and tissues [2, 3, 4]. Symptoms of ALD and the rate of progression are highly variable, even in individuals from the same family [5]. In some cases, ALD presents with a progressive leukodystrophy, known as cerebral ALD. This affects mostly boys < 12 years of age and is often fatal within 2–4 years, when left without hematopoietic stem cell transplantation [6]. In addition, all males and most females with ALD will develop a slowly progressive myelopathy [7, 8]. A common presenting endocrine symptom in boys and men with ALD is insufficient adrenocortical function, which eventually affects approximately 80% of male patients during life [9]. The occurrence of biochemical hypergonadotropic hypogonadism in patients with ALD is less well characterized but previous studies suggest there is an association. In this paper, the term hypogonadism refers specifically to hypergonadotropic hypogonadism, unless stated otherwise.

Testicular lesions in patients with ALD were first observed in 1974 [4]. In particular, the interstitial Leydig cells are affected, with intracytoplasmic lamellas and lamellar‐lipid inclusions in Leydig cells, as shown by electron microscope studies. These lesions are identical to those described in adrenocortical, Schwann, endoneurial, and microglial cells of ALD patients and pathognomonic for ALD [10]. Leydig cells are necessary for testosterone synthesis; therefore dysfunction of Leydig cells may eventually result in deficient testosterone production. In previous clinical studies, more than half of the male ALD patients reported diminished libido and/or erectile dysfunction [11, 12]. Diminished libido and erectile dysfunction might be attributed to testosterone deficiency, although these symptoms are also common symptoms of a myelopathy [13]. Damage to sympathetic or parasympathetic nerves in the spinal cord and peripheral ganglia can directly cause erectile dysfunction [14] and diminished libido could be a consequence of the erectile dysfunction and/or chronic disease in general [14, 15]. Distinguishing the causes of sexual symptoms (i.e., ALD‐induced myelopathy, hypogonadism, and/or related to having a chronic disease) is relevant since treatment options differ depending on the cause. In addition, it is important to establish the prevalence of sexual dysfunction and hypogonadism, since both are associated with significant consequences when untreated. Firstly, sexual dysfunction has been associated with psychological complaints [16] and is an under‐addressed problem since both doctors and patients are not likely to discuss it during routine outpatient visits [17]. Secondly, long‐term testosterone deficiency can lead to osteoporosis [18]. Testosterone replacement therapy is associated with improved bone mineral density, body composition, mood, and sexual desire [19, 20, 21].

The estimated prevalence of hypogonadism in ALD ranges from 0% to 12% [11, 12, 22]. However, only one study focused on plasma testosterone and gonadotropin levels for the diagnosis of hypogonadism, while correlations with clinical symptoms are lacking [22]. Moreover, all three studies used different cutoff values for testosterone and gonadotropins and different diagnostic methods for the analysis of gonadotropins were used, which makes accurate comparison complicated.

The aim of this observational study was to assess the prevalence of sexual dysfunction and its relationship to biochemical signs of hypogonadism and myelopathy in a large cohort of adult ALD males. This was done by interviewing and examining patients as well as measuring serum testosterone, luteinizing hormone (LH), and follicle‐stimulating hormone (FSH) levels. Secondly, the origin of the sexual dysfunction in patients with ALD was described.

## 2 Methods

### 2.1 Subjects

The current study was part of an ongoing prospective cohort study (The Dutch ALD cohort) at the Amsterdam University Medical Centers (location AMC, Amsterdam, The Netherlands) which serves as the national referral center for peroxisomal disorders and leukodystrophies. For the current study, all male ALD patients aged 18 years and over, under follow‐up at this center, were included. Patients with a history of hematopoietic stem cell transplantation because of cerebral ALD were excluded, since chemotherapeutics prior to the transplantation could have induced endocrine dysfunction. Patients were recruited at the outpatient neurology clinic between August 2020 and February 2021. Written informed consent was obtained from all participants and the project was approved by the local Institutional Review Board (IRB) (2018_310).

### 2.2 Clinical Assessment and Data Collection

Participants visited the outpatient endocrine and neurology clinics of the AMC. They were interviewed regarding the presence of sexual dysfunction by asking if they experienced erectile dysfunction or diminished libido. Medical history and medication use were also recorded. Furthermore, participants underwent a neurological examination. The severity of myelopathy was determined using the Expanded Disability Status Scale (EDSS) and the Severity Score System for Progressive Myelopathy (SSPROM). The EDSS, which was originally designed for multiple sclerosis, measures neurological disability on a scale of 0 (no disability) to 10 (death) [23]. SSPROM describes the severity of myelopathy and ranges from 0 to 100, with lower scores indicating more severe disease [24].

Patients who already used testosterone replacement therapy before the start of this study were included, but were not interviewed by an endocrinologist and no blood samples were taken since hypogonadism was already established in these patients.

### 2.3 Laboratory Tests

Morning blood samples were obtained after an overnight fast. For analysis of testosterone levels, serum was stored at 2°C–8°C for a maximum of 7 days or at −18°C to −22°C for a maximum of 2 months before analysis. LH and FSH were analyzed on the day of collection. Analyses of blood samples were performed at the Laboratory for Endocrinology, Amsterdam University Medical Centers (location AMC, Amsterdam, The Netherlands). Detection of testosterone was performed using tandem mass spectrometry. Concentrations of testosterone below 9 nmol/L were considered deficient [22]. The lower limit of quantification for testosterone was 0.1 nmol/L and the upper limit was 100 nmol/L. The within‐assay variation for testosterone was estimated at 3.2% for 4.7 nmol/L and at 1.6% for 25 nmol/L; the between‐assay variation was 1.5% and 2.6%, respectively.

Detection of LH and FSH concentrations was determined using non‐competitive immunoassays, at which the lower level of quantification was 0.1 IU/L for both gonadotropins. Within‐assay variations were estimated for LH at 2.3% (concentration: 1.2 U/L), 1.7% (5.9 U/L), 1.6% (19 U/L), and 1.6% (71 U/L). For FSH, within‐assay variations were 0.9% (1.5 U/L), 2.1% (4.9 U/L), 2.4% (15 U/L), and 3.2% (50 U/L). Reference values were based on the DELFIA hLH Spec kit version 13903238‐6 and DELFIA hFSH kit version 13903806‐3. LH values of 1.0–8.4 U/L and FSH values of 1.0–10.5 U/L were considered normal.

Based on the results of the endocrine tests, patients were divided into three groups: patients with (1) normal gonadal function (eugonadal), (2) biochemical subclinical hypogonadism, and (3) biochemical hypogonadism. Biochemically normal gonadal function was defined as testosterone, LH, and FSH levels within the normal range of the reference values. Biochemical subclinical hypogonadism applied to patients with normal testosterone levels but elevation of at least one of the gonadotropins. Biochemical hypogonadism was defined as testosterone levels below the lower limit of the reference value. A confirmed diagnosis of biochemical hypogonadism was defined as the combination of decreased testosterone level (below the lower limit of the reference value) and elevated gonadotropins.

In case of biochemical hypogonadism at the time of the outpatient visit, repeated measurements of morning testosterone and gonadotropins after overnight fasting would be planned according to the European Guidelines for diagnosis of hypogonadism in men [23]. This did, however, not apply as only one patient revealed biochemical hypogonadism but this patient could not visit the outpatient clinic again due to other illness.

### 2.4 Statistical Analysis

Descriptive statistics were used to illustrate the prevalence of sexual dysfunction and the three categories of biochemically established gonadal function. Patients were first grouped based on the presence (yes/no) of sexual dysfunction (erectile dysfunction and/or diminished libido). Between these groups, baseline characteristics (age, presence of adrenal insufficiency, and use of medication that could possibly cause sexual dysfunction), neurological status (as measured by EDSS and SSPROM), and biochemically established gonadal function were compared. To assess whether the variables age, interfering medication, and neurological function were significantly related to experiencing sexual dysfunction, multiple logistic regression analysis was used. In addition, the prevalence of biochemically normal gonadal function, biochemical subclinical hypogonadism, and biochemical hypogonadism in the total cohort was described, based on the laboratory definitions as described above. Continuous variables are expressed as means ± standard deviations or medians [interquartile ranges], where appropriate. Independent samples *t*‐tests (parametrical data) and Mann–Whitney *U* tests (non‐parametrical data) were used to analyze continuous data. Chi‐squared tests were used to analyze categorical data. R‐studio version 3.6.1 was used for analyses and a *p*‐value < 0.05 (two‐sided) was defined as statistically significant.

## 3 Results

### 3.1 Clinical and Endocrine Characteristics of the Cohort

The Dutch ALD cohort includes 62 male ALD patients. For this study, 14 patients were excluded (11 age < 18 years and 3 with a history of hematopoietic stem cell transplantation). Furthermore, as a result of the COVID‐19 pandemic, another 11 patients postponed their yearly visit. Therefore, 37 adult male ALD patients were included of whom 3 patients already received testosterone replacement therapy before the start of the study. Of these three patients we were able to retrieve data from the time of diagnosis of hypogonadism for one patient, but not for the other two patients. Nevertheless, we assumed the diagnosis was made correctly and classified all three patients as having hypogonadism. Additionally, during the study, one patient revealed having elevated testosterone levels and elevated gonadotropins. Since magnetic resonance imaging (MRI) showed a pituitary lesion and no other explanation for the elevated testosterone and gonadotropin levels was found, this patient was suspected of having a gonadotropinoma and therefore excluded from further analysis. As a consequence, in total 36 patients were included for analysis.

Patients' characteristics are presented in Table 1. Patients had a mean age of 42.0 (±15.7) years (ranging from 18 to 71 years). Adrenocortical insufficiency was present in 22 patients (61%). Median EDSS was 3.0 [IQR 0–6] and median SSPROM was 84 [IQR 78–100]. For the 33 patients without testosterone replacement therapy, the mean testosterone level was 17.2 (±6.0) nmol/L, median LH was 7.0 U/L (IQR 4.4–11.0), and median FSH was 6.8 U/L (IQR 4.2–9.6) (see Figure S1). Twelve patients (36%) used medication that could interfere with sexual function and/or endocrine laboratory measurements (see Table S1).

**TABLE 1:** Clinical characteristics and prevalence of sexual dysfunction.

	Total (*n* = 36)	Treated hypogonadism (*n* = 3)	Sexual dysfunction		*p*
			Yes (*n* = 18)	No (*n* = 15)	
Age in years, mean ± SD	42.0 (±15.7)	58.0 (±17.0)	45.9 (±12.8)	33.1 (±14.0)	< 0.01
Adrenocortical insufficiency, *n* (%)	22 (61)	1 (33)	10 (56)	11 (73)	0.35
Medication potentially interfering with sexual and/or gonadal functioning, *n* (%)	12 (36)^a^	NA	10 (56)	2 (13)	< 0.001
Severity of myelopathy					
EDSS, median [IQR]	3.0 [0–6]	6.5 [3.0–7.0]	4.8 [3–6]	0 [0–2]	< 0.001
SSPROM, median [IQR]	84 [78–100]	62.5 [62.0–85.0]	79.5 [69.9–83.8]	100 [90.3–100]	< 0.001
Testicular function					
Biochemically eugonadal, *n* (%)	20 (56)	NA	10 (55)	10 (67)	
Biochemical subclinical hypogonadism, *n* (%)	12 (33)	NA	7 (39)	5 (33)	
Biochemical hypogonadism, *n* (%)	4 (11)	3 (100)	1 (6)	NA	NA
Testosterone, mean ± SD (nmol/L)	17.3 (±6.6)	NA	17.7 (±7.1)	16.6 (±4.7)	0.64
LH, median [IQR] (U/L)	5.9 [4.0–9.5]	NA	7.3 [4.7–9.9]	5.3 [3.4–8.5]	0.24
FSH, median [IQR] (U/L)	6.3 [4.2–11.0]	NA	8.8 [5.8–11]	5.6 [4.4–10.2]	0.27
LH/FSH ratio, mean ± SD	0.9 (±0.5)	NA	1.0 (±0.6)	0.9 (±0.4)	0.80

Abbreviations: EDSS, Expanded Disability Status Scale; FSH, follicle‐stimulating hormone; LH, luteinizing hormone; NA, not applicable/available; SD, standard deviation; SSPROM, Severity Score System for Progressive Myelopathy.

^a^ *n* = 33 (participants without testosterone therapy).

### 3.2 Sexual Dysfunction and the Relation to Endocrine and Neurological Function

**FIGURE 1:**
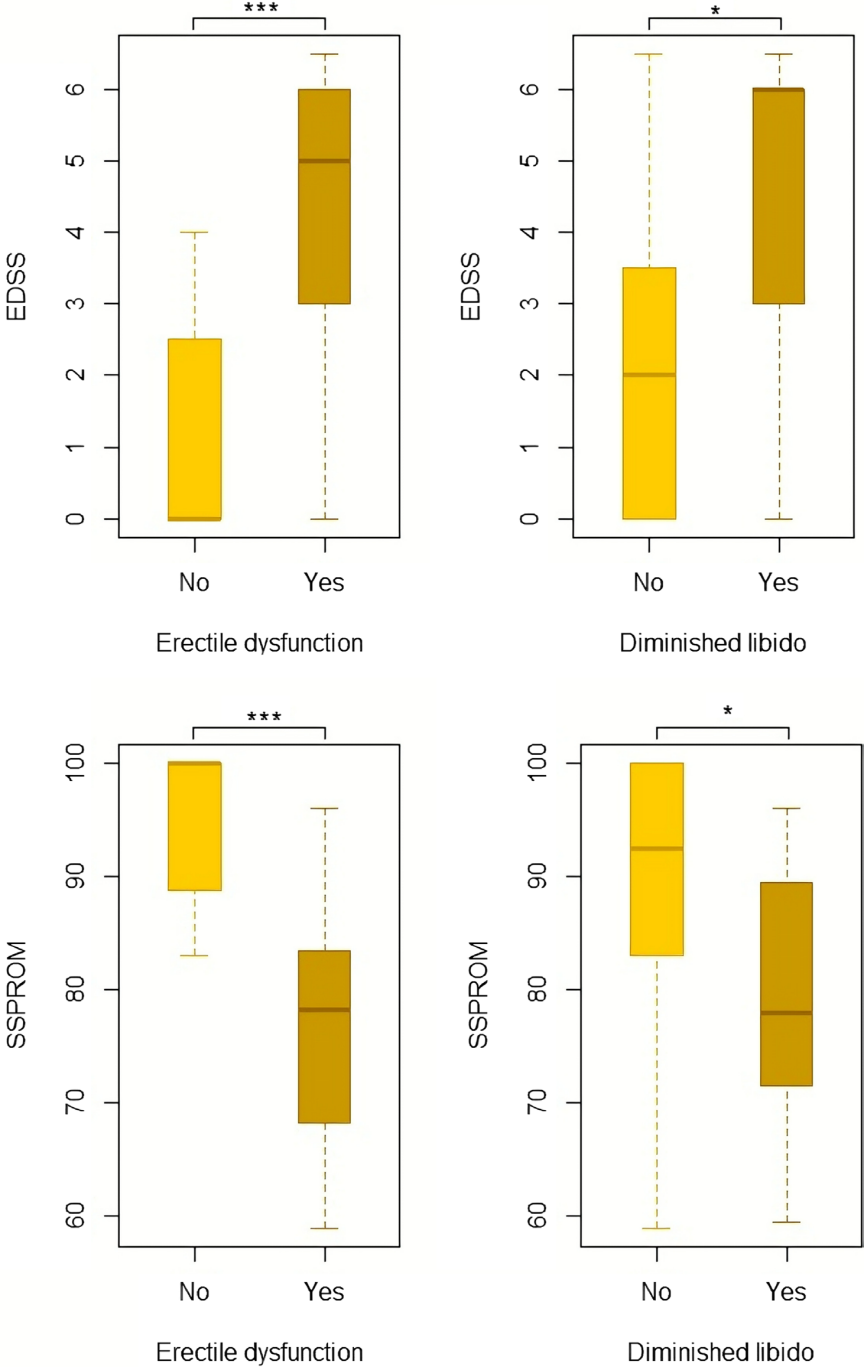
Presence of sexual dysfunction versus neurological function. Upper panel: (left) EDSS in patients with and without erectile dysfunction. (right) EDSS in patients with and without diminished libido. Lower panel: (left) SSPROM in patients with and without erectile dysfunction. (right) SSPROM in patients with and without diminished libido. Bars represent ranges (min–max) and boxes represent median with interquartile ranges. *p ≤ 0.05 and ***p < 0.001. EDSS, Expanded Disability Status Scale; SSPROM, Severity Score System for Progressive Myelopathy.

**FIGURE 2:**
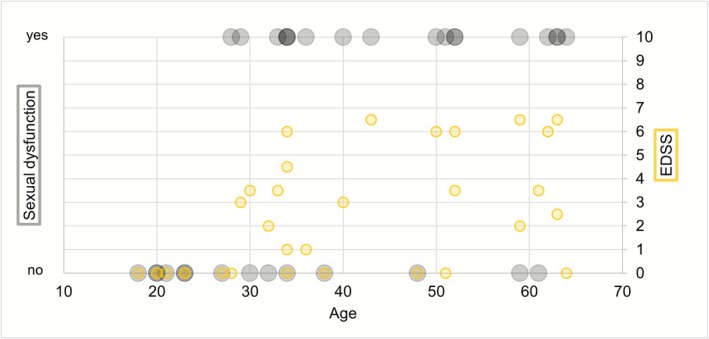
Distribution of neurological function and sexual dysfunction by age of participants. Gray: sexual dysfunction; yellow: EDSS (Expanded Disability Status Scale).

Complaints of sexual dysfunction were present in 18/33 (55%) patients. Seventeen patients experienced erectile dysfunction and 10 diminished libido. Nine patients with diminished libido also experienced erectile dysfunction. From the 18 males that experienced sexual dysfunction, 10 (55%) had normal gonadal function, 7 (39%) had biochemical subclinical hypogonadism, and 1 (6%) was biochemically diagnosed with hypogonadism (Table 1). Age and medication use were significantly different between patients with and without sexual dysfunction (Table 1). Patients with complaints of sexual dysfunction were older compared to patients without complaints of sexual dysfunction (45.9 (±12.8) vs. 33.1 (±14.0) years, respectively, *p* ≤ 0.001) and more often used medication with sexual dysfunction as a potential side effect (56% vs. 13%, *p* ≤ 0.001). In addition, EDSS and SSPROM differed significantly between patients with and without sexual dysfunction. Patients with sexual symptoms had higher EDSS and lower SSPROM (i.e., higher grade of impairment) compared to patients without sexual symptoms (EDSS: 4.8 (IQR 3–6) vs. 0.0 (IQR 0–2); *p* ≤ 0.001, and SSPROM: 79.5 (IQR 69.9–83.8) vs. 100 (IQR 90.3–100); *p* ≤ 0.001) (Table 1). The difference in neurological function remained significant after separating the sexual dysfunction into erectile dysfunction and diminished libido (Figure 1). In both logistic regression models (model 1: EDSS, interfering medication and age; model 2: SSPROM, interfering medication and age), neurological function was significantly related to experiencing sexual symptoms (model 1: *B* = 0.821, *p* = 0.004; model 2: *B* = −0.24, *p* = 0.003), interfering medication and age were not (*p* > 0.05). A distribution of sexual dysfunction and neurological function (EDSS) for every participant's age is shown in Figure 2.

### 3.3 Estimated Prevalence of (Subclinical) Hypogonadism

Following our laboratory definitions of groups, newly biochemically diagnosed hypogonadism was present in one 63‐year‐old male (testosterone: 5 nmol/L, LH: 14 U/L, FSH: 11 U/L). Together with the three patients already using testosterone replacement therapy, this results in a total of 4 out of 36 patients (11%) with (biochemical) hypogonadism (Table 1). Three of these four patients suffered from adrenal insufficiency and severe symptoms of myelopathy (EDSS 6–7); their age was between 34 and 69 years. One patient with biochemical hypogonadism had no adrenal insufficiency and only mild symptoms of myelopathy (EDSS 3.5), at the age of 72. Biochemically defined subclinical hypogonadism was present in 12/36 males (33%). In seven of these patients both gonadotropins were elevated and in only two out of seven patients LH levels were higher than FSH levels. Three patients had elevated LH only and two patients had elevated FSH only. Five patients with subclinical hypogonadism had no symptoms of sexual dysfunction. The remaining 20 males (56%) were considered biochemically eugonadal. Age between patients with normal gonadal function (41.0 ± 15.5 years) and biochemically defined subclinical hypogonadism (39.0 ± 12.3 years) did not differ significantly. Corticosteroids were used by seven patients (35%) in the biochemically eugonadal group, eight patients (66.7%) in the biochemical subclinical hypogonadism group, and two patients (50%) in the (biochemical) hypogonadism group (see Table S2) [24].

## 4 Discussion

This study shows that more than half (55%) of adult patients with ALD experience sexual dysfunction (i.e., erectile dysfunction and/or diminished libido). This is in accordance with previous studies by Brennemann et al. and Assies et al. who reported 53% and 58%, respectively [11, 12], and clearly illustrates this is a common problem in this population when compared to 31% in the general male population [25] It is important to recognize sexual dysfunction as it can be a major burden for patients and, depending on the cause, different forms of treatment are available.

We recognized two possible contributing factors to sexual dysfunction in this ALD patient population: neurological impairment caused by myelopathy and (biochemical) hypogonadism. Neurological impairment seems to be the main contributor to sexual dysfunction—especially erectile dysfunction—in ALD patients with complaints of sexual dysfunction. Patients experiencing sexual dysfunction had significantly more severe neurological impairment compared to those without sexual functioning complaints. Especially for the occurrence of erectile dysfunction, the difference in neurological impairment was considerable; median EDSS for patients with erectile dysfunction was 5.5 and SSPROM 78.3 (severe symptoms of myelopathy) compared to EDSS 0 and SSPROM 100 (no signs or symptoms of myelopathy) for patients without erectile dysfunction (Figure 1). For the occurrence of diminished libido, this difference was slightly smaller, although still significant. Moreover, neurological function (EDSS and SSPROM individually) remained significantly associated with experiencing sexual dysfunction after adjusting for age and use of possibly interfering medication. Interestingly, more than half (55%) of the patients with sexual dysfunction had biochemically normal gonadal function, indicating that in these patients, hypogonadism is not the main cause of their sexual dysfunction. Previous research reported that 38% of the females with symptomatic ALD experience sexual dysfunction, suggesting that the underlying mechanisms are complex and likely not solely determined by endocrine deficiencies [26].

The estimated prevalence of biochemical hypogonadism in this cohort was 11% (4/36; one newly diagnosed patient and three patients that already used testosterone replacement therapy prior to this study). This prevalence is close to the prevalence of 12% described by Assies et al. [11], but higher compared with the studies by Brennemann et al. and Stradomska et al., who both described a prevalence of 0% [12, 22]. This difference could be caused by differences in methodology for testosterone measurement, as this has improved over the past decades [27, 28, 29]. Moreover, Stradomska et al. used lower cutoff values for testosterone deficiency, which might have resulted in an underestimation of patients with hypogonadism. The prevalence of 11% in this cohort shows that hypogonadism should be recognized as part of the ALD spectrum, albeit in a minority of patients.

One third of males in this cohort (33%) had (slightly) abnormal gonadotropin levels but testosterone levels still within the normal range, meeting the criteria for subclinical hypogonadism (see Table 1). These results resemble other studies in the ALD field, in which (low) normal testosterone levels were found in combination with elevated LH or FSH (referred to as partial testicular insufficiency) [30, 31]. Interestingly, 2 out of 12 patients with a biochemical diagnosis of subclinical hypogonadism had elevated FSH levels only. In addition, 5 out of 7 patients with subclinical hypogonadism with elevation of both gonadotropins had higher FSH levels than LH levels. This suggests that besides the Leydig cell dysfunction as previously described [10], Sertoli cell dysfunction might also be in play.

In general, subclinical hypogonadism, regardless of its cause, is not accompanied by signs of sexual dysfunction. This is because positive feedback of the gonadotropins results in normal testosterone levels. Subclinical hypogonadism affects 9.5% of males in the general population, according to epidemiological data from the European Male Ageing Study, and the prevalence increases with age, up to 20% in males between 70 and 79 years of age [32]. Accordingly, in our study, the proportion of patients with biochemical subclinical hypogonadism actually experiencing sexual dysfunction was 39%, which was similar to the eugonadal group (55%). Age between these groups did not significantly differ. This indicates that partially (sub‐ or preclinical) affected testicular function is common in ALD, despite patients' age, but does not necessarily result in increased sexual dysfunction. There are no studies that show subclinical hypogonadism necessarily progresses into overt hypogonadism and testosterone therapy is therefore not recommended [32]. Prospective studies should be initiated to investigate whether and among which circumstances subclinical hypogonadism in ALD patients might progress into overt hypogonadism.

It should be noted that the number of patients using medication possibly interfering with sexual function was significantly higher in the group with sexual symptoms compared to the group without sexual symptoms. We could not conclude whether the sexual dysfunction was caused by use of this medication, as in most patients there was no temporal relation between the start of medication and the occurrence of symptoms. Moreover, the use of medication was not significantly independently related to experiencing sexual dysfunction in our regression models. Nevertheless, the use of medication should be taken into account when assessing sexual dysfunction, as discontinuation might improve or even resolve symptoms.

Based on our findings, it is advised to regularly discuss potential complaints of sexual dysfunction in all adult male ALD patients. When sexual dysfunction is present, testosterone and gonadotropin levels should be measured. In case of hypogonadism, patients should be further examined by an experienced endocrinologist to exclude other causes of hypogonadism through systematic evaluation. Testosterone supplementation should be considered based on guidelines in order to treat symptoms with careful consideration of potential benefits and risks such as possible increased risk for cardiovascular events in long‐term treatment [21]. Phosphodiesterase‐5 inhibitors (e.g., Sildenafil) might be considered in ALD patients with erectile dysfunction and normal testosterone levels. In case of subclinical hypogonadism, it is recommended to provide follow‐up to monitor whether or not patients develop overt hypogonadism, but treatment with testosterone replacement therapy is not recommended.

Some limitations should be taken into account when interpreting our results. As discussed before, hypogonadism was already diagnosed before the start of this study in 3 out of 4 patients and we could not retrieve all information regarding the diagnosis of hypogonadism. The prevalence of hypogonadism in this cohort (11%) could therefore be an overestimation, as we could not confirm if the diagnoses were made correctly. The one patient diagnosed with hypogonadism during the study suffered from other critical illnesses as well, which may worsen the experience of sexual symptoms and affect his testosterone level [33]. Moreover, other causes for primary hypogonadism could not be excluded as the patient was not able to visit the hospital for a follow‐up visit due to his illness. Furthermore, blood sampling in our study was supposed to be in the morning, because of the morning peak in testosterone levels as a result of the circadian rhythm of testosterone synthesis. Yet three patients were tested in the afternoon due to traveling time and timing of the appointment, including the patient that was diagnosed with hypogonadism. This might have resulted in an overestimation of the prevalence as reported in this study. Lastly, symptoms of sexual dysfunction were not assessed by using a validated questionnaire. A strength of this study is that for the detection of testosterone levels we used tandem mass spectrometry, which is much more sensitive compared to immunoassays used in previous research [34]. Also, we attributed cutoff values that are more strict compared to research estimating the prevalence of hypogonadism in the general population (< 9 nmol/L compared to < 10.4 nmol/L by Araujo et al.) [35], because the probability of symptoms (i.e., erectile dysfunction and diminished libido) increases with decreased testosterone levels (especially below 8–8.5 nmol/L) [36]. Therefore, the reported prevalence (11%) in this study might be a more reliable estimate as compared to previous studies.

In conclusion, this study shows that over half of the male ALD patients experience sexual dysfunction and neurological impairment plays an important causal role in these complaints. Overt hypogonadism does occur in ALD patients, though only in a minority. The prevalence of subclinical hypogonadism in this group of patients is high but it is unknown to which extent this results in overt hypogonadism. Therefore, these patients should be monitored over time. As both erectile dysfunction due to myelopathy as well as sexual dysfunction due to hypogonadism are well treatable, clinicians should actively discuss these problems in the yearly follow‐up care for ALD patients. Only when sexual dysfunction is present, testosterone and gonadotropins should be tested.

## Author Contributions

All authors substantially contributed to the work and were involved in (a) conception and design of the study and/or analysis and interpretation of data, and (b) revising the article critically for important intellectual content. All authors approved the final manuscript as submitted and agreed to be accountable for all aspects of the work. Stephanie I.W. van de Stadt, Aimy M.A. Wessel, Mirjam Langeveld, and Barbara Sjouke collected the data. Stephanie I.W. van de Stadt, Aimy M.A. Wessel, and Barbara Sjouke analyzed the data, wrote the first version of the manuscript, drafted, and wrote the final version of the manuscript. Stephanie I.W. van de Stadt, Mirjam Langeveld, Marc Engelen, and Barbara Sjouke initiated this study. All authors critically revised the manuscript for important intellectual content.

## Funding

This work was supported by Nederlandse Organisatie voor Wetenschappelijk Onderzoek (Grant No. 016196310).

## Ethics Statement

The study protocol was approved by the local Institutional Review Board (METC 2018‐013). All participants (and/or their legal guardians) gave written informed consent prior to participation.

## Conflicts of Interest

The authors declare no conflicts of interest.

## Supporting information


**Figure S1:** Testosterone, luteinizing hormone (LH), follicle‐stimulating hormone (FSH), and ratios of LH and FSH for participants with and without sexual dysfunction. Upper panel: (left) Testosterone of patients with and without sexual dysfunction. (right) Luteinizing hormone (LH) of patients with and without sexual dysfunction. Lower panel: (left) Follicle‐stimulating hormone (FSH) in patients with and without sexual dysfunction. (right) Ratio of LH and FSH of patients with and without sexual dysfunction. Bars represent ranges (min–max) and boxes represent median with interquartile ranges. Transparent bars represent normal ranges.

**Table S1:** Usage of medication potentially interfering with sexual and gonadal functioning.

**Table S2:** Percentage of patients using corticosteroids per groups based on biochemical gonadal status.

## Data Availability

The data that support the findings of this study are available from the corresponding author upon reasonable request.
