# Supplementary figures and images for: Sexual Dysfunction and Its Relationship With Hypogonadism and Myelopathy in Male Patients With X‐Linked Adrenoleukodystrophy
Source: J Inherit Metab Dis. 2025 Dec 2;49(1):e70121. doi: 10.1002/jimd.70121 (PMC12672194; doi:10.1002/jimd.70121)

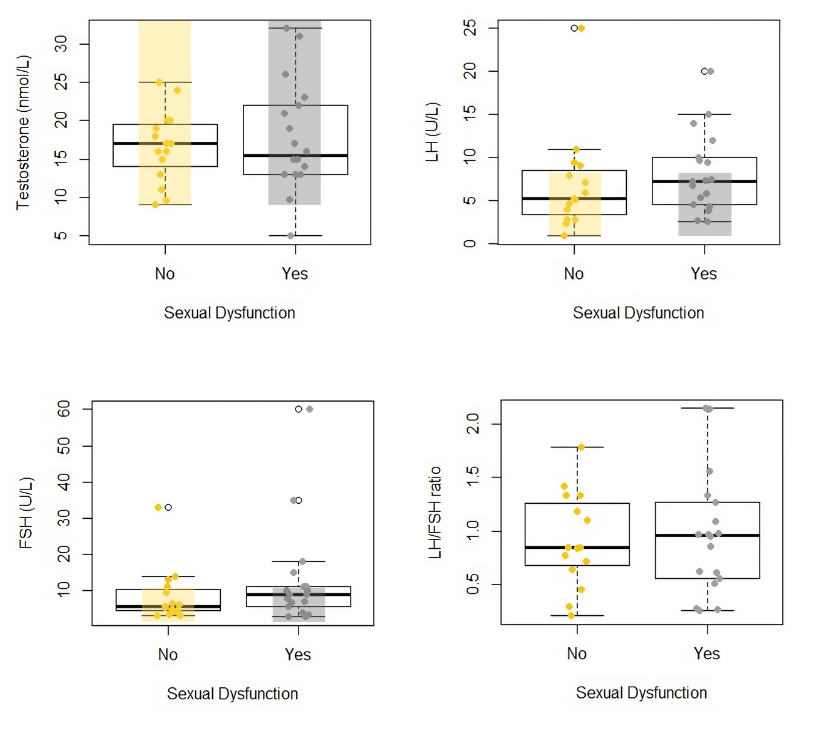

Supplement: Supplementary file 1 — Figure S1: Testosterone, luteinizing hormone (LH), follicle‐stimulating hormone (FSH), and ratios of LH and FSH for participants with and without sexual dysfunction. Upper panel: (left) Testosterone of patients with and without sexual dysfunction. (right) Luteinizing hormone (LH) of patients with and without sexual dysfunction. Lower panel: (left) Follicle‐stimulating hormone (FSH) in patients with and without sexual dysfunction. (right) Ratio of LH and FSH of patients with and without sexual dysfunction. Bars represent ranges (min–max) and boxes represent median with interquartile ranges. Transparent bars represent normal ranges. [file JIMD-49-0-s002.png]
