# Supplementary material for: Sexual Dysfunction and Its Relationship With Hypogonadism and Myelopathy in Male Patients With X‐Linked Adrenoleukodystrophy
Source: J Inherit Metab Dis. 2025 Dec 2;49(1):e70121. doi: 10.1002/jimd.70121 (PMC12672194; doi:10.1002/jimd.70121)
Supplement: Supplementary file 2 — Table S1: Usage of medication potentially interfering with sexual and gonadal functioning. [file JIMD-49-0-s001.docx]

**Supplemental Table 1. Usage of medication potentially interfering with sexual and gonadal functioning**

Shown are all patients with their sexual symptoms and the combinations of medication used with corresponding potential side-effects.

| Patient | Group ^a^ | Sexual Symptoms | Medication | Relevant potential  side-effect of medication  (frequency in general population) |
| --- | --- | --- | --- | --- |
| A | Eugonadal | ED | Amitriptyline | ED (1-10%) |
| B | Eugonadal | ED | Baclofen Carbamazepine Oxycodone | ED (0.01-0.1%) Sexual dysfunction (< 0.01%) ED or DL (0.1-1%), TD (unknown) |
| C | Eugonadal | ED | Perindopril | ED (0.1-1%) |
| D | Eugonadal | - | Simvastatin | ED (unknown) |
| E | Eugonadal | DL, ED | Oxybutynin | ED (< 0.01%) |
| F | Subclinical hypogonadism | DL, ED | Clonazepam  Fentanyl  Gabapentin  Gemfibrozil  Nifedipine  Oxycodone | DL and ED (unknown)  DL (< 0.01%)  Sexual dysfunction (unknown)  DL and ED (0.01-0.1%)  ED (0.1-1%)  ED or DL (0.1-1%), TD (unknown) |
| G | Subclinical hypogonadism | DL, ED | Baclofen | ED (0.01-0.1%) |
| H | Subclinical hypogonadism | - | Carbamazepine | Sexual dysfunction (< 0.01%) |
| I | Subclinical hypogonadism | DL, ED | Clonazepam | DL and ED (unknown) |
| J | Subclinical hypogonadism | ED | Metoprolol | ED (0.01-0.1%) |
| K | Subclinical hypogonadism | DL, ED | Pregabalin  Temazepam | ED and DL (1-10%)  DL (unknown) |
| L | Hypogonadism | DL, ED | Metoprolol | ED (0.01-0.1%) |

^a^ Based on biochemical classification as stated in the methods section.

Retrieved from Farmacotherapeutisch Kompas (Boomkamp et al. 2012)
ED: erectile dysfunction; DL: diminished libido; TD: testicular dysfunction
