# Supplementary material for: Sexual Dysfunction and Its Relationship With Hypogonadism and Myelopathy in Male Patients With X‐Linked Adrenoleukodystrophy
Source: J Inherit Metab Dis. 2025 Dec 2;49(1):e70121. doi: 10.1002/jimd.70121 (PMC12672194; doi:10.1002/jimd.70121)
Supplement: Supplementary file 3 — Table S2: Percentage of patients using corticosteroids per groups based on biochemical gonadal status. [file JIMD-49-0-s003.docx]

**Supplemental Table 2. Percentage of patients using corticosteroids per groups based on biochemical gonadal status.**

Shown are all the patients using corticosteroid suppletion.

|  | Eugonadal  (n=20) | Subclinical hypogonadism  (n=12) | Hypogonadism  (n=4) |
| --- | --- | --- | --- |
| Corticosteroids usage, n (%) | 7 (35) | 8 (66.7) | 2 (50) |
